# Effects of Fraxinellone on the Midgut Enzyme Activities of the 5th Instar Larvae of Oriental Armyworm, *Mythimna separata* Walker

**DOI:** 10.3390/toxins6092708

**Published:** 2014-09-11

**Authors:** Min Lv, Wenjun Wu, Huixia Liu

**Affiliations:** Institute of Pesticide Science, College of Plant Protection, Northwest A&F University, Yangling 712100, Shaanxi, China; E-Mails: wuwenjun@nwsuaf.edu.cn (W.W.); huixialiu@nwsuaf.edu.cn (H.L.)

**Keywords:** fraxinellone, digestive enzyme, detoxification enzyme, *Mythimna separata*

## Abstract

Isolated from *Dictamnus dasycarpus* Turcz., fraxinellone exhibited multiple bioactivities against insects. In the present paper, the changes of digestive enzymes and detoxification enzymes of *Mythimna separata* Walker (5th instar larvae), treated with fraxinellone, were investigated. Compared with those of the control, the α-amylase activity of the fraxinellone-treated 5th instar larvae was inhibited, whereas the level of their protease activity was increased. Based upon further studies on the specific proteases, the levels of the active alkaline trypsin-like enzyme (BA*p*NA as the substrate) and the chymotrypsin-like enzyme (BTEE as the substrate) activities of the treated larvae were declined; however, the level of activity of the weak alkaline trypsin-like enzyme (TAME as the substrate) of the treated ones was increased. Meanwhile, the activities of two detoxification enzymes, such as carboxylesterase (CarE) and glutathione S-transferase (GST), of the treated larvae were increased to some extent, but the activities of NADPH-P450 reductase and *O*-demethylase of the treated ones declined. Therefore, protease (especially the weak alkaline trypsin-like enzyme), CarE and GST played important roles in the metabolism of fraxinellone in the midgut of *Mythimna separata* (*M. separata*).

## 1. Introduction

Insect pest management is facing an economic and ecological challenge worldwide due to human and environmental hazards caused by the majority of synthetic pesticide chemicals. Identification of novel effective insecticidal compounds is essential to combating increasing resistance rates [1]. Botanicals containing active insecticidal phytochemicals play an important role in protecting plants from herbivores and appear to be promising in addressing some of these problems. Through diverse methods, such as acute toxicity, enzyme inhibition, and interference with the consumption of food [2], plant secondary metabolites are deleterious to herbivores.

**Figure 1:**
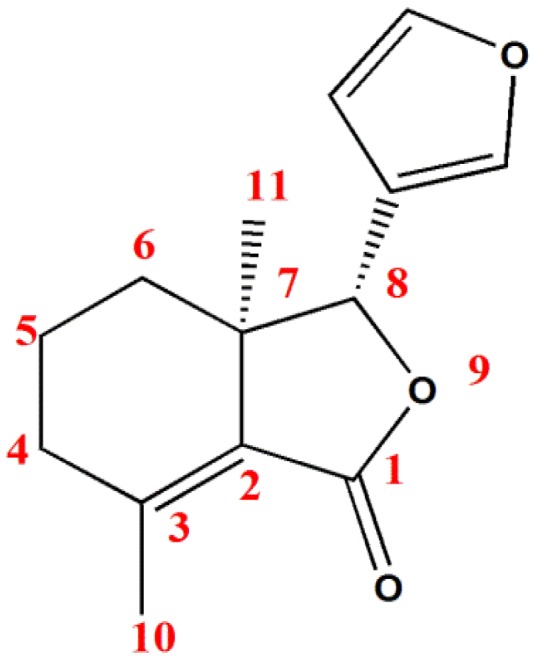
The chemical structure of fraxinellone.

Recently, fraxinellone (Figure 1) has been successfully isolated and identified from many species of Meliaceae and Rutaceae families (e.g., *Dictamnus. angustifolius* [3], *Fagaropsis glabra* [4], *Melia azadarach* [5,6], *Raulinoa echinata* [7,8], and *D. dasycarpus* [9,10]). In China, the dried root bark of *D. dasycarpus* Turcz., a traditional medicine, has been widely used for treatment of cough, rheumatism, skin diseases, and so on. In 1978, fraxinellone was for the first time isolated from the plants of genus Dictamnus, *D. angustifolius* [11]. Okamura *et al.* in 1997 [12] and Heasley in 2011 [13] reported the total synthesis of fraxinellone, respectively. Meanwhile, fraxinellone exhibited many activities, such as antifertility activity, antiplatelet aggregation, vascular relaxing activity [14,15], fungicidal activity [16], and insecticidal activity [16]. Especially, fraxinellone exhibited a delayed stomach poison, and feeding deterrent activities against pests. For example, Liu *et al.* [9] found that fraxinellone displayed significant feeding deterrence against two stored-product insects such as *Tribolium castaneum* Herbst and *Sitophilus zeamais* Motsch. Against adults and larvae of *T. castaneum* and adults of *S. zeamais*, the EC_50_ values of fraxinellone were 36.4, 29.1, and 71.2 ppm, respectively. Similarly, against adults and larvae of *T. castaneum*, Asian corn borer, *Ostrinia furnacalis* [17] and tobacco budworm, *Heliothis virescens* [18], their growth rates and food consumption were obviously inhibited by fraxinellone. In our previous study, fraxinellone could also inhibit the larval development of oriental armyworm, *Mythimna separata* Walker. Against the 3rd instar larvae of *M. separata*, the LC_50_ value of fraxinellone was 15.95 mg·mL^−1^ [19]. Moreover, further histopathology research revealed that the organelles of *M. separata* midgut were changed clearly after treatment with fraxinellone [20].

It is well-known that digestive enzymes and detoxification enzymes of the insect midgut are involved in the detoxification or metabolism of xenobiotics [21]. Plant defenses against insect herbivores are partially mediated by enzymes that weaken digestive processes in the insect gut. Protease, α-amylase and lipase play an important role in the digestive of food in the gut. On the other hand, carboxylesterase (CarE), cytochrome P450 (P450) and glutathione S-transferase (GST) are three kinds of important multi-functional enzymes involved in the metabolism of a broad range of xenobiotics, such as drugs, pesticides and plant toxins in animals. 

The larvae of *M. separata* Walker, a typical lepidopteran pest, is widely distributed in China, Japan, Southeast Asia, India, Eastern Australia, New Zealand and some Pacific Islands, and attacks many agricultural plant species such as maize, sorghum and rice. Sometimes, its outbreaks result in widespread incidence and complete crop loss [22]. Recently, development of new pesticides originated from plant secondary metabolites has received much research attention. In the present paper, the changes of digestive enzymes (α-amylase, lipase, total protease and specific protease) and detoxification enzymes (CarE, GST and P450) of *M. separata* (5th instar larvae) treated by fraxinellone were investigated. Moreover, the relationship between the metabolism of fraxinellone and the above two kinds of midgut enzymes was also discussed.

## 2. Results and Discussion

### 2.1. The Effects of Fraxinellone on the Midgut Digestive Enzymes

**Figure 2:**
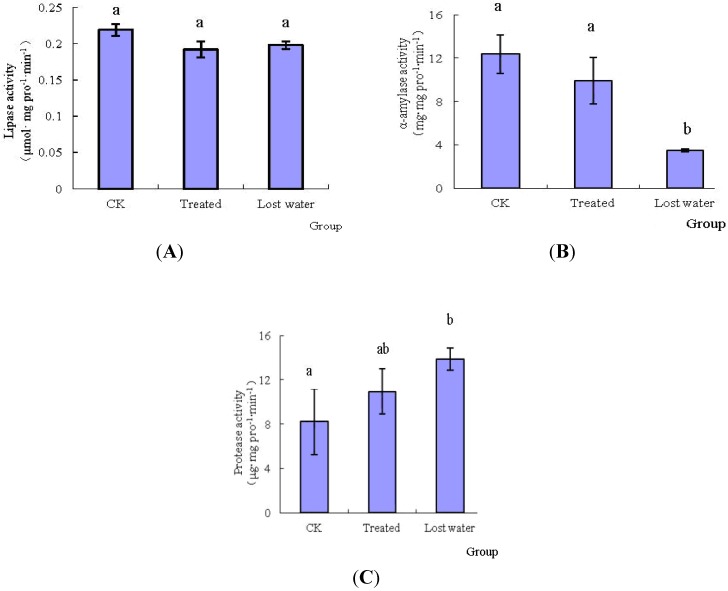
Effects of fraxinellone on the activities of digestive enzymes. The uppercase letters of (**A**), (**B**) and (**C**) were represented the activities of lipase, α-amylase and non-specificprotease, respectively. Data in the figure are mean ± SE. Different lowercase letters indicate the significant differences among different groups (*p* < 0.05). The meanings of letter are the same in the following figures.

After treatment with fraxinellone, the activities of midgut digestive enzymes of *M. separata* are shown in Figure 2. Compared with those of the control group of *M. separata*, fraxinellone inhibited the α-amylase, and the inhibition ratios of α-amylase activities in no-symptom larvae and lost-water larvae were 19.98% and 71.80%, respectively. Meanwhile, it has no distinct effect on lipase. In contrast, in the period of lost water, the level of non-specific protease activity was increased by 68% than that of the control, and the difference between them was obviously observed. That is, the non-specific protease perhaps played a key role in the catabolism process of fraxinellone in the midgut of *M. separata*.

### 2.2. The Effects of Fraxinellone on the Specific Protease Activities

**Figure 3:**
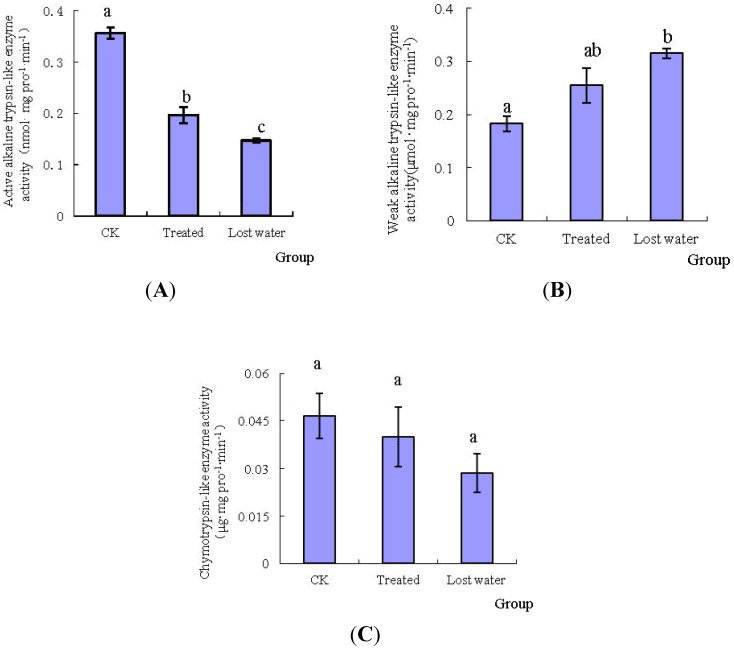
Effects of fraxinellone on the activities of specific proteases. The uppercase letters of (**A**), (**B**) and (**C**) were represented the activities of active alkaline trypsin-like enzyme, weak alkaline trypsin-like enzyme and chymotrypsin-like enzyme, respectively.

After treatment with fraxinellone, the activities of midgut specific protease of *M. separata* are shown in Figure 3. Based upon further studies on the specific proteases, the levels of the active alkaline trypsin-like enzyme (BApNA as the substrate) and the chymotrypsin-like enzyme (BTEE as the substrate) activities of the treated larvae were declined; however, the level of activity of the weak alkaline trypsin-like enzyme (TAME as the substrate) of the treated ones was increased. For example, during the period of lost water, the activities of the above two enzymes were only 41.31% and 61.72% of those of the control. Whereas, as compared with those of the control, the activities of weak alkaline trypsin-like enzyme of no-symptom and lost-water larvae, which was increased 0.396-fold and 0.727-fold, respectively, were enhanced after treatment with fraxinellone.

### 2.3. The Effects of Fraxinellone on the Midgut Detoxification Enzymes

**Figure 4:**
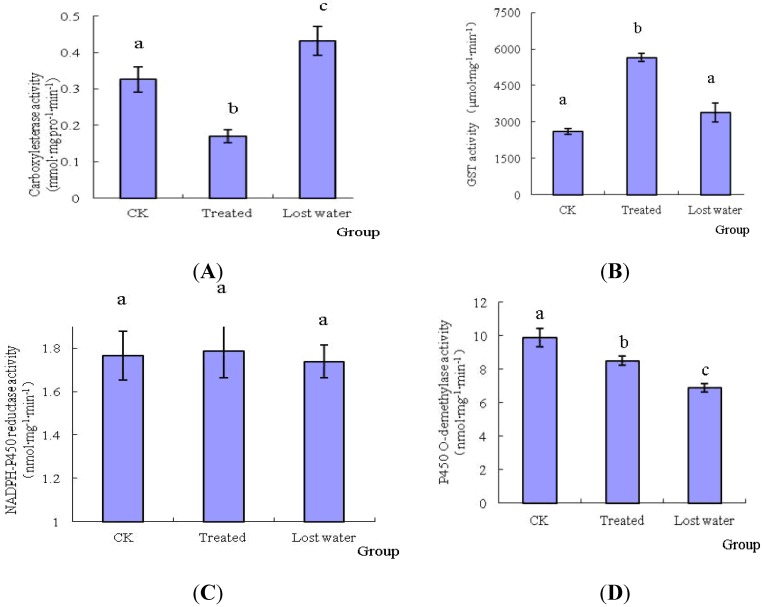
Effects of fraxinellone onthe activities of detoxification enzymes. The uppercase letters of (**A**), (**B**), (**C**) and (**D**) were represented the activities of CarE, GST, NADPH-P450 reductase, and P450 *O*-demethylase, respectively.

After treatment with fraxinellone, the activities of midgut detoxification enzymes of *M. separata* are shown in Figure 4. The activities of NADPH-P450 reductase had no evident changes. However, the activities of P450 *O*-demethylase were inhibited by fraxinellone. For example, the inhibition ratio of P450 *O*-demethylase was 30.29% at the lost water period. Interestingly, the activities of two detoxification enzymes, such as CarE and GST, of the treated 5th instar larvae were increased to some extent. Therefore, CarE and GST played an important role in the metabolism of fraxinellone in the midgut of *M. separata*.

Incorporated into artificial diets, fraxinellone significantly reduced the activities of a-amylase and non-specific proteases, but an increased level of cytochrome P450s was observed in the larval midguts of *O. furnacalis* [17]. In this paper, the activities of α-amylase, lipase, and cytochrome P450 were inhibited by fraxinellone, but the activities of protease (especially the weak alkaline trypsin-like enzyme), carboxylesterase and glutathione S-transferase were increased. The proteinases are a major group of hydrolytic enzymes in insects and are involved in digestive processes. Serine and cysteine proteinases are the two major proteinase classes in insects [23]. Because the pH value of the gut of *M. separata* were alkaline, *M. separata* were capable of protecting themselves by inhibiting the bind of protein and tannin even if they ingested plants containing tannic acid such as wheat or cereal leaves. The larvae of *M. separata* ingested some dosage of fraxinellone, and finally died. It suggested that the feeding deterrent activity of fraxinellone resulted in the extreme starvation of *M. separata*, meanwhile, too excessive protease, CarE and GST were secreted or synthesized to protect themselves from the damage. Moreover, in our previous paper, we found the poisoned insects exhibited typical stomach poisoned symptoms (e.g., vomit and diarrhea), and fraxinellone showed delayed insecticidal activity against *M. separata*. Histopathology research demonstrated that the organelles of *M. separata* midgut were obviously changed after treatment with fraxinellone [20]. The changed organelles will affect the secretion of enzymes.

P450 is the hemoproteins encoded by a superfamily of genes nearly ubiquitously distributed in different organisms from all biological kingdoms. The reactions catalyzed were extremely diverse [24]. Herein we only determined the activities of NADPH-P450 reductase and *O*-demethylase, but the other components of P450, such as the P450 content, were still not involved. Therefore, further related studies will be carried out in our lab.

## 3. Experimental Section

### 3.1. Insects

The laboratory-adapted *M. separata* Walker was obtained from Institute of Pesticide Science, Northwest A&F University (NWAFU) (Yangling, China). The strain had been reared on wheat leaves under laboratory conditions for about 20 years, and was never in contact with insecticides. 

### 3.2. Chemicals

Fraxinellone (>98%) was provided by Institute of Pesticide Science, NWAFU, and diluted with acetone to required dosages. Eserine, *p*-nitroanisole (*p*-NA), cytochrome C, GSH, NADPH, CDNB, BA*p*NA, TAME and BTEE were purchased from Sigma Chemical Co. Ltd. (Shanghai, China).

### 3.3. Enzyme Preparation

Two-day-old 5th instar larvae of *M. separata* were treated with fraxinellone by means of stomach poison. Technical grade fraxinellone was diluted to 20 mg·mL^−1^ in acetone. Leaf disks were treated with 1 μL diluted sample. The control groups were fed with leaf disks treated with acetone only. Fraxinellone showed the delayed insecticidal activity. After several hours, the poisoned larvae of *M. separata* lost water [20]. Then, the control, treated (no symptom), lost water larvae of *M. separata* were selected, respectively.

Used for digestive enzyme, the midguts were removed from the dissected larvae and their contents were rinsed in ice-cold 0.15 M NaCl. Midguts were homogenized in homogenization buffer containing 1 mL 20% glycerol, the homogenate was centrifuged at 1 × 10^4^ g for 20 min. Prepared for carboxylesterase, the midguts were homogenized in 0.04 M sodium phosphate buffer (pH 7.0), and were centrifuged at 1.5 × 10^4^ g for 20 min. For GST activity assay, the midguts were homogenized in 0.1 M sodium phosphate homogenization buffer (pH 7.5) containing 15% glycerol, 2 mM EDTA. The homogenate was centrifuged at 1 × 10^4^ g for 20 min. To measure the activity of cytochrome P450, midguts were homogenized in 0.1 M sodium phosphate homogenization buffer (pH 7.5) containing 10% glycerol, 1 mM PMSF, 1 mM PTU, 1 mM EDTA. The homogenate was centrifuged at 1 × 10^4^ g for 20 min. All of the supernatant was decanted into a clean tube and used immediately for the related assay. The whole process was carried out at 0–4 °C.

### 3.4. Digestive Enzyme Assays

The α-amylase activity was assayed by the method detailed by Feng *et al.* [25]. Maltobiose was used for the preparation of the standard curve. The reaction system including supernatant, starch solution and buffer, was incubated for 20 min at 30 °C. Then 0.4 N NaOH was added to the above reaction system to stop the reaction. Finally, a chromogenic reagent was used to measure the absorbance at 520 nm.

The lipase activity was assayed by the method detailed by Chen [26]. Triglyceride (TG) was used for the preparation of the standard curve. The reaction system including supernatant, Tris-HCl buffer and olive oil solution, was incubated for 20 min at 37 °C, and then the absorbance was measured at 420 nm.

The non-specific protease activity was assayed by the method detailed by Wang and Qin [27]. Tyrosine was used for the preparation of the standard curve. The reaction system including supernatant, casein solution and phosphate buffer, was incubated them for 20 min at 30 °C. Then, trichloroacetic acid solution and Folin’s phonel reagent A and B were added to the above reaction system. Then, the absorbance was measured at 680 nm.

The activities of specific protease were assayed by the method detailed by Zhang *et al.* [28]. BA*p*NA and TAME were used as the substrates to measure the activities of active and weak alkaline trypsin-like enzymes, while BTEE was used as the substrate to the chymotrypsin-like enzyme. The extinction coefficients of BA*p*NA, TAME, and BTEE were 1.05 × 10^4^, 540, and 964 M^−1^·cm^−1^, respectively.

### 3.5. Detoxification Enzyme Assays

Carboxylesterase activity was assayed by the method of Liang *et al.* [29]. The reaction system included supernatant, α-naphthyl acetate (containing 0.3 mM^−1^ eserine) and Fast Blue RR salt. The assay was run for 15 min at 595 nm. α-Naphthol was used for the preparation of standard curve.

GST activity was assayed by the method of Habig *et al.* [30], and 1-chloro-2,4-dinitrobenzene (CDNB) was used as the substrate. The reaction system included supernatant, CDNB and glutathione. The reaction was measured for 5 min at 334 nm at room temperature. An extinction coefficient of 9.5 mM^−1^·cm^−1^ was used to determine the activity of GST.

NADPH P450-reductase activity was assayed by the method of Yasukochi and Masters [31]. The reaction system included supernatant, cytochrome C, NADPH, and sodium phosphate buffer. The reaction was measured for 3 min at 550 nm at room temperature. An extinction coefficient of 21.1 mM^−1^·cm^−1^ was used to determine the activity of P450 reductase.

Cytochrome P450 *O*-demethylase activity was assayed by the method of Qiu *et al.* [32]. The reaction system included supernatant, *p*-nitroanisole, NADPH and sodium phosphate buffer. After shaking 30 min, HCl solution was add to the above reaction system to stop the reaction. Then the product, *p*-Nitrophenol, was extracted with CHCl_3_ and centrifuged at 3000 rpm for 15 min. The CHCl_3_ fraction was back-extracted with NaOH solution. Finally, the absorbance of NaOH solution was recorded at 405 nm. *p*-Nitrophenol was used for the preparation of standard curve.

### 3.6. Protein Assay

The estimation of protein content was done using the method detailed by Bradford [33]. Absorbance was converted to protein concentration by analysis against a simultaneously determined standard curve of bovine serum albumin.

### 3.7. Statistical Analysis

All the assay data of enzyme activities were analyzed by Duncan’s posttest, which was conducted using SPSS 10.0 software to examine the significance of differences.

## 4. Conclusions

In summary, the changes of digestive enzymes and detoxification enzymes of *M. separata* (5th instar larvae) treated by fraxinellone were studied. It was demonstrated that protease (especially the weak alkaline trypsin-like enzyme) CarE and GST played important roles in the metabolism of fraxinellone in the midgut of *Mythimna separata* Walker. The present results will lay a foundation for further research of action mechanism of fraxinellone, and will develop it as a new insecticidal agent in the future.
